# Identification of potential serum biomarkers for breast cancer using a functional proteomics technology

**DOI:** 10.1186/s40364-017-0092-9

**Published:** 2017-03-14

**Authors:** David L. Wang, Chuanguang Xiao, Guofeng Fu, Xing Wang, Liang Li

**Affiliations:** 1 Department of Biology, Vanderbilt University, Nashville, TN USA; 2 Zibo Central Hospital, Zibo, China; 3 Array Bridge Inc., 4320 Forest Park Ave, Suite 303, St. Louis, MO 63108 USA

**Keywords:** Biomarkers, Diagnostic kits, Breast cancer, Functional proteomics, Enzyme activity, Hexokinases, Proteases, Protein Elution Plate

## Abstract

**Background:**

Cancer is a genetic disease; its development and metastasis depend on the function of many proteins. Human serum contains thousands of proteins; it is a window for the homeostasis of individual’s health. Many of the proteins found in the human serum could be potential biomarkers for cancer early detection and drug efficacy evaluation.

**Methods:**

In this study, a functional proteomics technology was used to systematically monitor metabolic enzyme and protease activities from resolved serum proteins produced by a modified 2-D gel separation and subsequent Protein Elution Plate, a method collectively called PEP. All the experiments were repeated at least twice to ensure the validity of the findings.

**Results:**

For the first time, significant differences were found between breast cancer patient serum and normal serum in two families of enzymes known to be involved in cancer development and metastasis: metabolic enzymes and proteases. Multiple enzyme species were identified in the serum assayed directly or after enrichment. Both qualitative and quantitative differences in the metabolic enzyme and protease activity were detected between breast cancer patient and control group, providing excellent biomarker candidates for breast cancer diagnosis and drug development.

**Conclusions:**

This study identified several potential functional protein biomarkers from breast cancer patient serum. It also demonstrated that the functional proteomics technology, PEP, can be applied to the analysis of any functional proteins in human serum which contains thousands of proteins. The study indicated that the functional domain of the human serum could be unlocked with the PEP technology, pointing to a novel alternative for the development of diagnosis biomarkers for breast cancer and other diseases.

## Background

Human breast cancer is a major cause of morbidity and mortality in women. Data from the International Agency for Research on Cancer (IARC) showed that the incidence of cancer has increased all over the world. Regarding breast cancer, the highest incidence rates were found in the United States and Western Europe with 101 and 85 new cases per 100.000 women, respectively [1]. The American Cancer Society estimates that each year more than 230,000 Americans women will be diagnosed with this neoplasia and that more than 40,000 will die of the disease in the United States. Presymptomatic screening to detect early-stage cancer while it is still resectable with potential for cure can significantly reduce breast cancer-related mortality. Unfortunately, only about half of the breast cancers diagnosed are before the metastatic stage [2]. One reason that contributes to the poor prognosis of patients diagnosed with breast cancer is the fact that the diagnosis is often delayed due to limitations in mammography. Screen-film mammography (SFM) is considered the gold standard for breast cancer screening and detection. However, its optimal performance is only observed among women over 50 years old. Also, SFM has other limitations such as high rates of false-negative (between 4 and 34%). In addition, there is a high rate of false-positives that lead to unnecessary biopsy procedures [1].

In the last decade, many new technologies have been utilized for biomarker discovery with significant progress. Each of these technologies has focused on a different type of biological entity such as circulating tumor cells (CTC), extracellular vesicles, micro-RNAs and cancer-derived cell-free DNA or circulating tumor-derived DNA (ctDNA) [3–12]. However, several fundamental issues such as tumor heterogeneity, plasticity and diversity of cancer stem cells (CSC) make biomarker discovery and development a challenging endeavor. The variation introduced during sample collection and storage and the lack of robust validation approach once biomarker leads are identified further complicate biomarker development. As a result of these hurdles, there are currently no United States FDA-approved serum tests for early detection of the disease [1]. Given the considerable public health importance of breast cancer, it is crucial to quickly identify new biomarkers with the potential to enhance early diagnosis and to predict patient prognosis, drug resistance development and treatment choice.

Blood based biomarkers have great potential in cancer screening and their role could extend further from general population risk assessment to treatment response evaluation and recurrence monitoring. The rich content of diverse cellular and molecular elements in blood, which provide information about the health status of an individual, make it an ideal compartment to develop noninvasive diagnostics for cancer [13]. However, despite a large literature collection related to biomarkers for common cancers, blood based diagnostic tests that inform about the presence of cancer at an early stage and predict treatment response have been difficult to develop [11, 14–16].

For the past decade, proteomics has been used for the discovery of potential biomarkers from human fluids including serum [4, 6, 17, 18]. So far, most efforts in proteomics has been focused on the identification and sequence annotation of the proteome by mass spectrometry analyses of peptides derived through proteolytic processing of the parent proteome [19]. In such a manner, thousands of proteins have been identified from human serum (www.serumproteome.org). However, no validated protein biomarker currently exists for use in routine clinical practice for breast cancer early detection, prognosis and the prediction of treatment response. It is generally recognized that sequence annotation alone cannot capture this vital information, so new strategies are necessary.

Two-Dimensional (2-D) Gel Electrophoresis is a powerful tool used to separate complex protein samples because more than 10,000 protein spots can be detected with information on their relative abundance and post-translational modifications. Recently, a modified 2-D Gel Electrophoresis process was integrated with a special protein elution and refolding process to achieve high resolution of protein species from a proteome called PEP [20]. Many of the fractions recovered by the PEP technology with enzyme activity appear to contain just one or two major proteins, making the positive identification of the protein of interest relatively easy.

It is hypothesized that the levels and distributions of certain enzyme functions in serum could produce proteomic features and collective profiles which reflect physiological changes of an individual and can serve as possible biomarkers or diagnostic parameters. Our earlier studies using lung cancer patient serum and normal serum have identified many fractions with metabolic enzyme activity and the enzymes identified could serve as potential functional biomarkers for the diagnostic of lung cancer [21]. In the current study, we used both enriched serum samples as well as original serum pools from breast cancer patients and normal people for the systematic analysis of metabolic enzyme and protease activities with the PEP technology. In both type of samples, a large number of fractions with metabolic enzymes and proteases were identified with significant differences between breast cancer and normal serum. We believe that the further identification and validation of those functional proteins could lead to the development of biomarkers for breast diagnosis.

## Methods

### Materials

All the chemicals were purchased from MilliporeSigma (St. Louis, MO). Isoelectric Focusing (IEF) unit that is capable of running IEF at different length is from Bio-Rad (PROTEAN IEF Cell). Spectrophotometer Plate Reader capable of reading 384-well plates with a wide wavelength selection and fluorescence reading is the SPECTRAMax Plus from Molecular Devices (Sunnydale, CA). Semi-Blot unit for protein transfer such as Bio-Rad’s Trans-Blot SD Semi-Dry Transfer Cell. AlbuVoid™ serum protein enrichment beads was from Biotech Support Group (Monmouth Junction, NJ). Protein Elution Plate (PEP) is a product of Array Bridge (St. Louis, MO).

### AlbuVoid™ treatment for low abundance serum protein enrichment

200 mg of AlbuVoid™ beads were used to process 0.8 ml of human serum (contains about 40 mg total serum protein). The breast cancer patient serum and the matching normal people serum were collected at Zibo Central Hospital in China after the approval from the Hospital Ethics Committee with reference number of 20140102. Serum samples from normal people or breast cancer patient were pooled with equal volume (100 μl each) respectively and either used directly in the analysis or enriched for low abundance serum proteins with AlbuVoid™ according to the manufacturer’s instruction before loading to the IEF. The enriched low abundance serum proteins were eluted with 0.8 ml elution solution containing 8 M urea, 2% CHAPS in 25 mM phosphate buffer, pH 8.0. The protein concentration was determined by BCA before 2-D gel electrophoresis.

### Isoelectric focusing (IEF) and 2-D gel electrophoresis

To prepare for the IEF separation, Bio-Lyte Ampholyte (Bio-Rad #1631112) was added to the serum solution directly or AlbuVoid™ elute above with a final concentration of 0.5%. Rehydration was using 0.4 ml sample solution with nonlinear pH 3–10 11 cm IPG strip (Bio-Rad ReadyStrip #1632016) overnight with a total loading of 1 mg protein/IPG strip. In the experiments without AlbuVoid™ enrichment, 1 mg of serum protein from the pooled breast cancer patient or unaffected individual was used directly following the same sample preparation as described above. All the enriched and unenriched serum proteins were separated in the same IEF run. The proteins were separated using the following setting: 0–7000 linear gradient voltage for 4 h hold at 7000 voltages overnight until running termination at room temperature. After IEF, the IPG strips were taken off the running unit, mineral oil from the IPG strip was absorbed with a paper towel and the IPG strip was transferred to a 12-lane refolding tray (Bio-Rad #1654025). 4 ml refolding solution was added to each lane with the IPG strip and incubated for 10 min., which allows the urea to diffuse out of the IPG strip and permits the refolding of the protein., This was followed by incubation in electrophoresis transfer buffer (Tris-glycine with 0.1% SDS), which allows for the further diffusion of urea from the IPG strip and the binding of SDS to the protein so that all the proteins were negatively charged. For protein refolding, a proprietary protein refolding solution was used; the solution contains multiple metal elements to replace the possible loss of metal ions as enzyme cofactors. A redox system to mimic the cell cytoplasm was used to assist the protein refolding process. After protein refolding, the IPG strip was laid down to a precast 2-D gel (Bio-Rad 10–20% Criterion Gel #3450107) with the acidic end of the IPG on the left side of the 2-D gel when facing the gel apparatus. The gel was operated at 80 V for 15 min. followed by running at 120 voltages until the dye front of the gel was 0.5 cm from the bottom edge of the gel.

### Electroelution and protein recovery from the Protein Elution Plate (PEP)

After second dimension gel electrophoresis, the gel was taken out from the cassette, and laid on top of the PEP plate which was filled with elution solution. The proteins were transferred from the gel to the PEP plate for 60 min. at 20 V using a Semi-Blot apparatus from Bio-Rad (#1703940.). After protein transfer, the gel was carefully lifted from the PEP plate, and a multi-channel pipette transferred the eluted proteins from the PEP plate to a master plate which contained 50 μl PBS in each well. About 40–45 μl of solution could be transferred from the PEP plate to the Master Plate for a total volume of 90–95 μl in each well. In this analysis, 25 μl solutions was taken from each well in the Master Plate and transferred to an enzyme assay plate to perform the enzyme assay.

### Hexokinase activity assay

Hexokinase activity can be monitored by a cascade reaction as follows:

$$ \begin{array}{l}\mathrm{Substrates}\ \mathrm{added}\left\{\mathrm{D}\hbox{-} \mathrm{Glucose} + \mathrm{ATP}\right\}\overset{\mathrm{Hexokinase}}{\to}\mathrm{Products}\left\{\mathrm{D}\hbox{-} \mathrm{Glucose}\ 6\hbox{-} \mathrm{Phosphate} + \mathrm{ADP}\right\}\\ {}\mathrm{D}\hbox{-} \mathrm{Glucose}\ 6\hbox{-} \mathrm{Phosphate} + \mathrm{\ss}\hbox{-} \mathrm{NADP}\overset{\mathrm{G}\hbox{-} 6\hbox{-} \mathrm{PDH}}{\to }6\hbox{-} \mathrm{Phospho}\hbox{-} \mathrm{D}\hbox{-} \mathrm{Gluconate} + \mathrm{\ss}\hbox{-} \mathrm{NADP}\mathrm{H}\end{array} $$

In the final assay solution, glucose was at 216 mM; MgCl2 at 7.8 mM, ATP at 0.74 mM and NADP at 1.1 mM. 25 μl of this enzyme assay solution was mixed with 25 μl of sample from the Master Plate (described above) and the enzyme activity was monitored by the 340 nm absorbance from the reduction of NADP to NADPH. The readings at 0, 1 h., 2 h. was recorded for both the normal serum and breast cancer patient serum sample. However, in lieu of purified G-6-PDH, 0.25 mg/ml beef liver protein was used as the source of Glucose-6-Phosphate Dehydrogenase (G-6-PDH). The assay thus reports the additive contributions of the endogenous hexokinase activity present in the beef liver extract, and any exogenous activity from the presence of test sera protein in the PEP plate, which may influence the reduction of NAD or NADP (the reporting signal). In light of the ambiguities that may arise from such a reporting system, the primary goal of this investigation was to generate sufficient signal intensities and activity features which could monitored and compared between the two samples types within an ‘omics’ context. Therefore, this broader spectrum assay was chosen that could potentially detect the activities of downstream glycolytic and other cross-regulating proteins from the test sera.

### Protease activity

FITC-labeled casein was used as general protease substrate at 0.5 mg/mL final concentration.

25 μl each of the PEP plate sample and substrate were incubated at room temperature overnight in the dark, after protease digestion, the casein was precipitated with 10% TCA (trichloroacetic acid) and the supernatant was neutralized with Tris base and used for the fluorescence measurement.

Proteases Assay:

$$ \mathrm{Casein}\ \left(\mathrm{substrate}\right)\hbox{-} \mathrm{FITC}\overset{\mathrm{Proteases}}{\to}\mathrm{Hydrolyzed}\ \mathrm{Casein} + \mathrm{FITC} $$

### Enzyme activity display

Two Microsoft Excel formats were used to display the enzyme activities. One is to use the 3-D column display and the other is to use the heat map.

## Results

### Comparison of hexokinase activity from normal serum and breast cancer patient serum

**Fig. 1:**
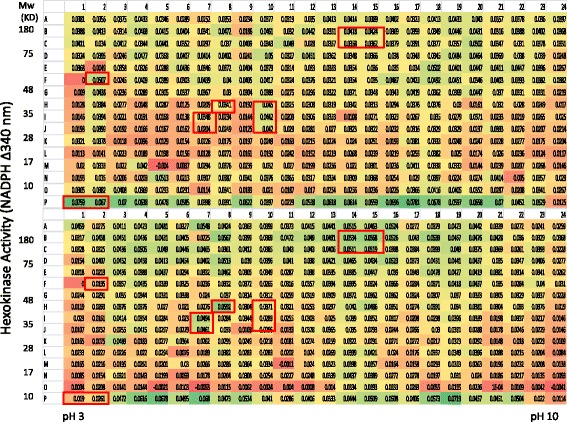
Direct measurement of hexokinase activity from normal and breast cancer serum after PEP protein separation and elution (60 min). After elution of proteins from the 2-D gel into the 384-well PEP plate, the eluted proteins were further transferred into a 384-well Master Plate with 50 μl refolding solution. 25 μl of the solution from the Master Plate was further transferred into a 384-well Enzyme Assay plate, 25 μl of hexokinase assay components were was added to each well, the increase of OD 340 nm by NADP reduction was measured in a spectrophotometer, the OD readings were taken at 0 and 60 min. the OD340 nm increase from 60 min. was calculated by subtracting the value from the 0 min. reading. Boxed fractions indicated samples of interest

**Fig. 2:**
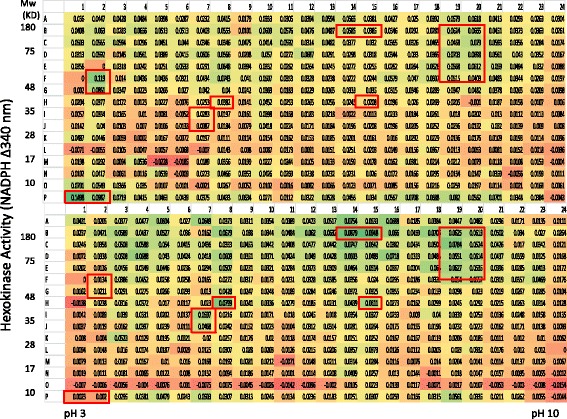
Direct measurement of hexokinase activity from normal and breast cancer serum after PEP protein separation and elution (120 min). The conditions were the same as in Fig. 1, the OD readings were taken at 0 and 120 min. the OD340 nm increase from 120 min. was calculated by subtracting the value from the 0 min. reading. Boxed fractions indicated samples of interest

Previous effort in proteomics has identified thousands of proteins from human serum, a high percentage of the proteins identified are enzymes with significant numbers belongs to metabolic enzymes and proteases (www.serumproteome.org). There have been many reports of single or multiple protein panels as potential biomarkers for breast cancer diagnosis, however so far there are no routinely used serum-based biomarkers approved for breast cancer [1]. As a successful diagnostic biomarker, it will need to achieve a high level of sensitivity and specificity in its detection to minimize false negatives and false positives respectively because a high proportion of those false results have significant consequences both economically and also emotionally. During the development of serum biomarkers, one of the challenges is the wide range of physiological variations among the general population which causes potential biomarkers to overlap between the normal and patient group. In retrospective studies, a panel of serum biomarkers are often identified with excellent separation between the normal and disease group but failed during the validation process using collected clinical samples. In the current study, two aspects were unique: 1. the search for possible biomarkers is from a new domain of the serum proteome, i.e. the functional domain. It is hoped that this new dimension of information can provide a distinct signature for breast cancer when compared with normal serum. 2. When selecting the biomarker candidates, the effort was started with pooled serum instead of individuals. In this approach, the individual physiological variations of serum proteins from the normal or cancer patients were averaged, which will partially reduce the variations from the individuals and help identify those proteins that showed significant differences between the normal and disease groups. Once the potential biomarker(s) are identified, their discriminating power for the normal and disease individuals will be tested during the validation stage. 3. Only those fractions with qualitative differences (the detected active fraction is totally missing from a group) or 10-fold differences will be further investigated. This will increase the possibility of eventual validation of the biomarker candida(s) in a large clinical sample collection. To demonstrate that hexokinase activity can be monitored from the PEP fractions, the reduction of NADP was measured at different time points. As can be seen in Figs. 1 and 2, many fractions from the normal serum have time-dependent hexokinase activities. Similarly, measurements on the serum from breast cancer patients also showed many fractions with hexokinase activities, and more interestingly, there are both qualitative and quantitative differences between the normal serum and cancer patient serum. For example, in the normal serum, F2 and G2 have significant levels of hexokinase activity whereas the corresponding fractions in the breast cancer patient serum are at baseline levels; the same is true for fractions P1 and P2. Conversely, from the breast cancer patient serum, fractions K3, I7, J7 and H8 each have significant hexokinase activities while the enzyme activity from the corresponding fractions in the normal group only showed baseline levels of activity (Fig. 2). As expected, those fractions with enzyme activity showed a time-dependent activity increase (Figs. 1 and 2). Another area that showed a significant difference in hexokinase activity was the high molecular weight region with pI between 7 and 8 (see the boxed area in Fig. 2). Interestingly, the fractions with hexokinase activity were detected across a wide range of molecular size and isoelectric points, suggesting that: 1.) there are many serum proteins that could directly or indirectly impact hexokinase activities within this assay system, and 2.) there could be protein variants that show different hexokinase activity among the fractions.

### Comparison of protease activity from normal serum and breast cancer patient serum

**Fig. 3:**
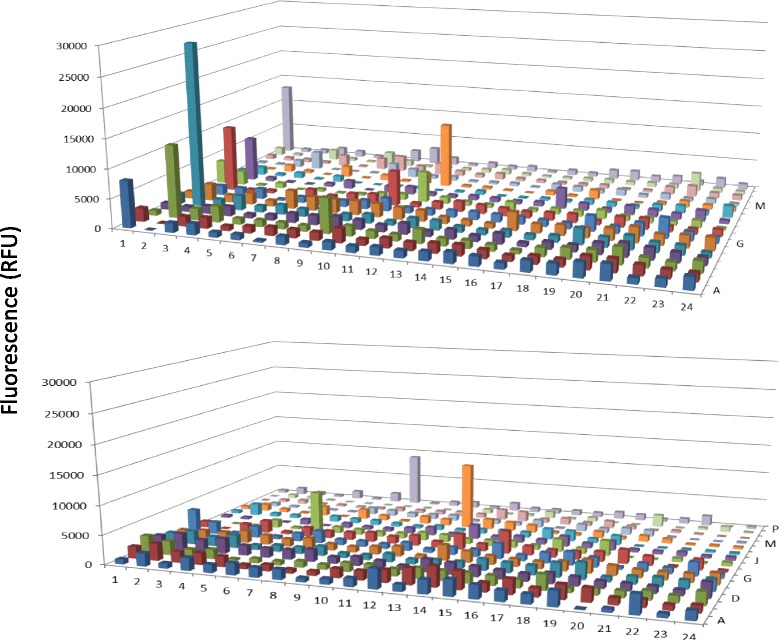
Direct measurement of protease activity from normal and breast cancer serum after PEP protein separation and elution. The conditions were the same as in Fig. 1 except protease substrate was added to the assay plate. The assay was carried out at room temperature overnight, for more details please refer to the material and method. Boxed fractions indicated samples of interest

Previous studies have shown that a large number of proteases exist in the human serum. (www.serumproteome.org) However, only a limited number of proteases were shown to have enzymatic activities [22]. In this study, protease activities were monitored systematically from the human serum directly using the PEP technology. Multiple fractions with protease activities were identified that showed significant different between normal and breast cancer patient serum (Fig. 3). The majority of the fractions with protease activities were detected in the acidic protein region (pI from 3 to 7) whereas very little activity was observed in the basic protein region. For protease activity detection, protein enrichment with AlbuVoid™ will make a significant difference, the principle of AlbuVoid™ is to use a chemically synthesized material to absorb serum proteins except albumin, by allowing albumin to be specifically wash off from the AlbuVoid™ column, the other low abundance serum proteins are enriched on the column and eluted with an elution solution ; details of the protease activity analysis will be discussed in the later part of this section.

### Comparison of hexokinase activity from enriched normal serum and breast cancer patient serum

**Fig. 4:**
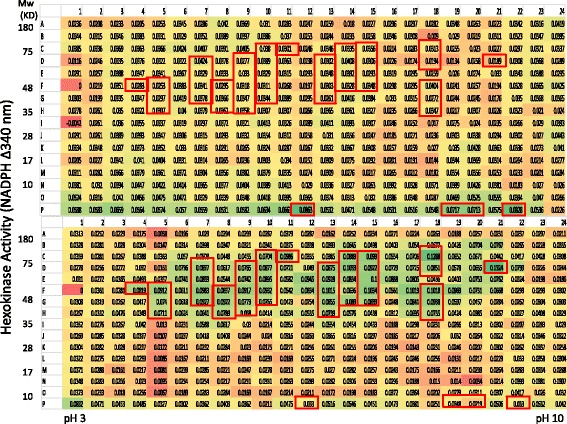
Measurement of hexokinase activity from normal and breast cancer serum after AlbuVoid™ enrichment and PEP protein separation and elution (60 min). 800 μg AlbuVoid-enriched serum proteins from normal group was loaded onto an IPG strip and separated by IEF. After further separation by SDS-PAGE, the proteins were eluted into the PEP plate and hexokinase activities were analyzed from each of the 384 wells. Hexokinase activity was measured by NADP reduction at 340 nm at 0 and 60 min, the OD340 nm increase from 60 min. was calculated by subtracting the value from the 0 min. reading. Boxed fractions indicated samples of interest

**Fig. 5:**
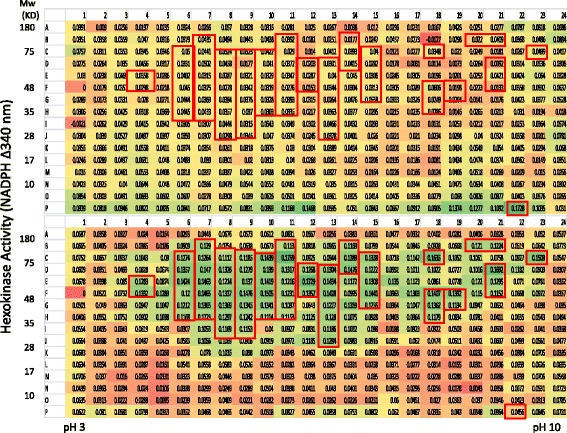
Measurement of hexokinase activity from normal and breast cancer serum after AlbuVoid™ enrichment and PEP protein separation and elution (120 min). The conditions were the same as in Fig. 4, hexokinase activity was measured by NADP reduction at 340 nm at 0 and 120 min, the OD340 nm increase from 120 min. was calculated by subtracting the value from the 0 min. reading. Boxed fractions indicated samples of interest

Previous work in using AlbuVoid™ has demonstrated that the profile of serum proteins and enzymatic activities will be changed significantly during the enrichment [21, 23]. (In this study, it was shown that the depletion of albumin can significantly enrich hexokinase activities (Figs. 4 and 5). Compared with the direct serum assay (Figs. 1 and 2), more fractions with hexokinase activities were detected across a wide range of molecular weight and isoelectric points (see Fig. 5 boxed). In the comparison of normal and breast cancer patient serum, many fractions with both qualitative and quantitative hexokinase activity differences were detected (see the boxed fractions in Fig. 5 for details). This demonstrated that AlbuVoid™ is a very effective tool for the enrichment of low abundancy proteins with enzyme activity. As a result of this enrichment, there are more candidate fractions to choose from for further biological validation as potential biomarkers.

### Comparison of protease activity from enriched normal serum and breast cancer patient serum

**Fig. 6:**
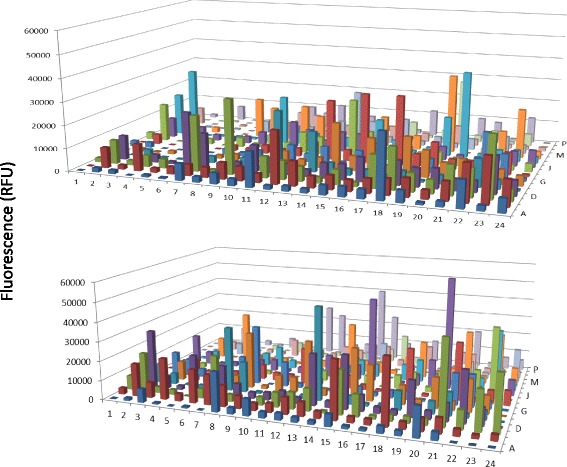
Protease activity from normal and breast cancer serum after AlbuVoid™ enrichment and PEP protein separation and elution. The conditions were the same as in Fig. 3 except the testing sample is from AlbuVoid™ enrichment. The assay was carried out at room temperature overnight, for more details please refer to the material and method. Boxed fractions indicated samples of interest

The most dramatic difference for enzyme activity detection in using the AlbuVoid™ for serum protein enrichment was demonstrated in the case of protease activity analysis. Figure 6 indicated that a large number of fractions were shown to have protease activities after serum protein enrichment. Compared with the direct serum proteinase measurement (Fig. 3), both the levels and species of proteases were increased significantly in the enriched serum sample. The fact that very few fractions were detected with protease activity in the direct serum analysis suggested that the protease levels in the serum were below the detection threshold of protease activity method used in this study, and it is necessary to use AlbuVoid™ to enrich these low level proteases to bring them to a high enough level to be detected.

## Discussion

In US, breast cancer is the most commonly diagnosed malignancies and the leading cause of cancer mortality among women. Approximately 40,000 deaths from the disease occur annually in the US. Worldwide, more than 450,000 deaths occur each year with 1,300,000 diagnosed [24]. The main factor that contributes to breast cancer mortality is the presence of metastasis. Of those diagnosed with breast cancer, 30 to 85% of the patients already have bone metastases and the median survival rate after diagnosis is 25 to 72 months. Human serum is one of the bodily health windows from which the homeostasis of the body can be monitored [13].

In human serum, more than 10,000 proteins have been detected and thousands of them have been identified by mass spectrometry. However, there is a bottleneck in the further use of those serum proteins as potential biomarkers: the clinical validation of the biomarker candidates that meet the requirements of both sensitivity and specificity. Given the cost of developing useful assays such as monoclonal antibody-based immunoassays, it is not practical to test the hypothesis on thousands of serum proteins in a clinical setting. Therefore, different or more efficient approaches should be considered in the efforts for breast cancer biomarker discovery. Significant progress has been made in the field of breast cancer biomarker discovery in the past decade; however, the early accurate detection of the disease remains a challenge [1, 2, 9, 10, 12, 25, 26]. In a recent review of potential biomarkers for breast cancer, fifteen biomarkers with demonstrated promise in initial studies were reported two of them are for diagnosis and the rest are for prognosis [1]. In addition to protein based biomarker candidates, miRNA and other nucleic acid-based breast cancer biomarkers were also reported recently but no validated biomarker currently exists for use in routine clinical practice

It has long been recognized that cancer has significantly different metabolic behavior when compared with normal cells. Otto Warburg was the first to report the increased metabolic glucose activity in cancer tissue [27, 28], and there has been many reports linking increased metabolic activities with cancer development in the past few years [29–36]. As a result of extensive studies on cancer metabolism, many compounds targeting various metabolic enzymes are in different stages of clinical trials with some encouraging efficacy. Up to now, most of the efforts in serum breast cancer biomarker discovery has been focused on the level of proteins or protein post-translational modifications [3–5, 13, 15, 37]. The goal is to find a protein-based signature for the disease has only been met with limited success. Since the human serum reflects the homeostasis of the body, we hypothesized that among the thousands of proteins identified, metabolic enzymes and other functional proteins may provide a unique window to look into the breast cancer status. Recently, a functional proteomics technology was developed that can analyze the function of serum proteins systematically after 2-D gel electrophoresis [20]. Using this technology, it was demonstrated that the activities of many metabolic enzymes could be monitored from mouse cochleae tissue after drug treatment, and that proteins with differential regulation could be identified directly by mass spectrometry because each fraction recovered in the PEP technology contained just one or a few proteins, In a further study with human lung cancer serum, it was shown that many fractions with metabolic enzyme activities could be detected from lung cancer patient serum [21]. In this further study, it was demonstrated that many fractions with metabolic enzyme activities or protease activities could be detected from normal and breast cancer patient serum with or without AlbuVoidTM^.^ protein enrichment. Comparison between the direct serum analysis and the enriched serum analysis showed that the number of active fractions and levels of enzymatic activities were significantly increased after protein enrichment. This is not surprising given the high protein concentration of the serum protein and the earlier finding that human albumin is associated with many species of serum proteins. First of all, since the principle of AlbuVoid™ serum low abundance protein enrichment is through avoiding the binding of albumin to the column whereas proteins other than albumin will bind any proteins with similar biochemical properties as albumin could be in the flow-through fraction. Secondly, as mentioned above, since human albumin was found to be associated with large amount of low abundance proteins, the avoidance of albumin would also exclude those proteins that bind to albumin. In spite of the impact on the serum protein composition, the AlbuVoid™ enrichment is still a very effective method to enrich low abundance proteins from human serum [23]. This is especially important for certain functional enzymes because the detection and quantitation of enzyme activity is proportional to the quantity of the enzyme present and many enzymes may be below the detection threshold of the assay limit. This was the case in the protease activity assay as more and different enzyme fractions were detected with the protein enrichment (Figs. 3 and 6). It should be pointed out that in the current study, the hexokinase assay system was designed to detect any rate-limiting enzymes in the glycolytic pathway because the first enzyme activity in the pathway, hexokinase, was selected and beef liver extract was used as the source of supporting enzymes for the assay. This design was intended to detect as many active metabolic enzymes from the serum as possible. Therefore, any glycolytic enzymes downstream of hexokinase could potentially enhance hexokinase activity by removing downstream products from the system. Because of this assay design, any downstream enzymes in the metabolic pathway could contribute to the detected activity. Previous studies have identified a large number of proteases from human serum through mass spectrometry and a few proteases have been shown to possess catalytic activity (www.serumproteome.org). In this study, a large number of fractions with protease activity were detected from human serum, and many differences were identified between normal and cancer patient serum (Figs. 3 and 6). As expected, there are more species of proteases being detected in the enriched serum samples as compared with the direct serum assay and the enzyme activity levels for many fractions were also significantly higher in the enriched samples. This suggested that for the protease activity assay used in the study, many of the serum proteases were below the detection or quantitation threshold, and the use of an effective serum protein method is critical to reveal the important information in the functional aspects of proteins in the serum. In the earlier studies, several other enzymes have been tested in the PEP platform for serum proteins including protein kinases, alkaline phosphatases, NADH or NADPH-dependent oxidases and GAPDH [23]. In all the enzymes analyzed, multiple fractions were detected with enzymatic activities. Given the fact that most of the cellular proteins have variants because of post-translational modifications, it is possible that only one or a few protein variants released by the cancer cells and survived in the serum could retain a high correlation with human diseases.

## Conclusions

In this study, multiple functional fractions with biomarker candidates were identified for the first time from breast cancer serum using the PEP technology. It is hoped that the combination of methods for serum protein enrichment and PEP technology, which allows functional proteins to be molecularly profiled and compared, can help discover new breast cancer biomarkers. Furthermore, these same technologies can easily be extended for the development of biomarkers, drug targets or diagnostic kits for other types of cancer or diseases.

## Abbreviation

PEP: Protein Elution Plate
